# The first long-lived mutants: discovery of the insulin/IGF-1 pathway for ageing

**DOI:** 10.1098/rstb.2010.0276

**Published:** 2011-01-12

**Authors:** Cynthia Kenyon

**Affiliations:** University of California, San Francisco, CA 94158, USA

**Keywords:** *daf-2*, *daf-16*, FOXO, history, insulin, IFG-1

## Abstract

Inhibiting insulin/IGF-1 signalling extends lifespan and delays age-related disease in species throughout the animal kingdom. This life-extension pathway, the first to be defined, was discovered through genetic studies in the small roundworm *Caenorhabditis elegans*. This discovery is described here.

This article describes the discovery of a genetic pathway that regulates ageing. In spite of the fascinating qualities of the ageing process, such as its remarkably different pace in different species, until the last few decades ageing was not thought to be subject to any active regulation. Now we know that the rate of ageing is indeed subject to regulation, by classical signalling pathways. These pathways link the ageing rate to environmental and physiological cues, and may even underlie its diversification during evolution. At the heart of these pathways are stress and metabolic sensors such as insulin and IGF-1 hormones, TOR kinase and AMP kinase, whose up- or downregulation can trigger a variety of cell-protective mechanisms that extend lifespan.

The first lifespan pathway to be discovered was the insulin/IGF-1/FOXO pathway. This pathway is evolutionarily conserved: mutations in many insulin and IGF-1-pathway genes extend the lifespan of mammals and several have been linked to human longevity. In particular, DNA variants in FOXO transcription-factor genes have been linked to exceptional longevity in human cohorts from around the world. These and other exciting findings grew out of basic genetic research in the small nematode *Caenorhabditis elegans*, where the first long-lived mutants were isolated. In this article, I describe how some of these discoveries came about. I am not a historian but rather a scientist who played a role in these discoveries. Although the literature I cite establishes an objective narrative of these findings, I will describe the studies in which I participated from my own personal perspective, being part of the story as it unfolded.

*Caenorhabditis elegans* lives for only a few weeks, but during its lifetime it undergoes a physical and behavioural decline that anyone, even someone who has never seen the worm, can recognize as ageing (see electronic supplementary material, online movie). I remember realizing this for the first time in the early 1980s when I was a postdoctoral fellow with Sydney Brenner in Cambridge, UK, and I was cultivating a mutant strain that had very few progeny. Normal *C. elegans* hermaphrodites produce 300 self-progeny during their first week of life. So a single worm on a culture dish soon disappears into a sea of progeny and cannot be found. I left culture dishes with my almost-infertile mutants in the incubator for several weeks, and then looked at them. With so few progeny, the original animals were still easy to find, and to my surprise, they looked old. This concept, that worms get old, really struck me. I sat there, feeling a little sorry for them, and then wondered whether there were genes that controlled ageing and how one might find them.

In fact, around that time, Michael Klass was already screening for long-lived mutants. Klass was a postdoctoral in David Hirsh's laboratory at the University of Colorado, Boulder, USA. His elegant early work set the stage for genetic studies. Klass showed, for example, that *C. elegans* lives longer and has fewer progeny when subjected to dietary restriction. This phenomenon had first been observed in rodents in the 1930s and had remained unexplained. Klass also showed that worms, which are ectotherms, live longer at low temperature than at high temperature (Klass 1977) [1]. By doing temperature-shift experiments, he discovered that the animals carry a memory of their childhood temperature that affects their adult lifespan, a phenomenon that has yet to be explained molecularly. Then, to find genes affecting ageing, Klass carried out a screen for long-lived mutants, noting that ‘Because many mutations in vital genes will lead to a decrease in lifespan, it is potentially more interesting to obtain mutants with significantly increased life spans.’ (Klass 1983) [2, p. 279] (also see Johnson & Wood 1982 [3]). Klass mutagenized a group of animals and looked among their second-generation descendants for mutants that lived long. To curtail reproduction while also ensuring that any long-lived mutants could be propagated, he carried out his screen in animals harbouring a temperature-sensitive *fer-15* mutation, which prevented reproduction at high temperature. He established little families from several hundred individual potential mutants at low (permissive) temperature, and then tested members of each family at high temperature, where they could not reproduce, to ask which families, if any, were long lived. Eight of Klass' families were long-lived, but, after observing their additional phenotypes, he concluded that they probably did not harbour interesting lifespan mutations. For example, several mutants were feeding-defective, and Klass concluded that these animals were probably long-lived because of dietary restriction. He wrote ‘The high correlation of the decreased rate of food ingestion of these mutants with their increased longevity is interpreted as indicating that the increased longevity is most likely due to reduced caloric intake. These results appear to indicate that specific lifespan genes are extremely rare or, alternatively, lifespan is controlled in a polygenic fashion’ (Klass 1983) [2, p. 279].

Klass' mutants were not abandoned, however. Another researcher, Tom Johnson, continued to study them. Previously, Tom had been exploring the genetic basis of ageing in another way (Johnson & Wood 1982; Johnson 1987) [3,4]. Working in Bill Wood's laboratory, which was also at the University of Colorado, Tom had crossed two different strains of worms, the normal laboratory strain Bristol and a French strain with a similar lifespan called Bergerac, and established new ‘recombinant-inbred’ lines from their descendants. These different lines should contain new combinations of polymorphic alleles present in the parental Bristol or Bergerac strains. Tom found something very interesting: some recombinant-inbred lines lived much longer than others. This meant that there were genetic polymorphisms in these strains that could lengthen or shorten lifespan.

Subsequently, Tom outcrossed some of Klass' mutants and recovered a strain that ate perfectly well and yet still lived long. He wrote: ‘*age-1*(*hx546*) is a recessive mutant allele in *Caenorhabditis elegans* that results in an increase in mean lifespan averaging 40% … at 20 degrees; at 25 degrees *age-1*(*hx546*) averages a 65% increase in mean lifespan … ’ (Friedman & Johnson 1988) [5, p. 75]. This was a very interesting finding. However, the mutant displayed a phenotype that raised the possibility of a more trivial explanation for its longevity: it had sharply reduced fertility. Evolutionary theory predicted that animals with reduced fertility would live longer, as they would be able to divert resources that would otherwise be used for reproduction into somatic maintenance. Thus it was possible that the primary effect of *age-1*(*hx546*) was to affect fertility, not ageing. In Johnson's words: ‘*age-1*(*hx546*) is associated with a 75% decrease in hermaphrodite self-fertility … It is likely that the action of *age-1* in lengthening life results not from eliminating a programmed ageing function but rather from reduced hermaphrodite self-fertility or from some other unknown metabolic or physiologic alteration.’ (Friedman & Johnson 1988) [5, p. 75]. Subsequently, Johnson went on to characterize the phenotype of this mutant in more detail, showing, for example, that *age-1*(*hx546*) slows the exponential increase in mortality rate that occurs with age (Johnson 1990) [6]. Interestingly, later, in 1993, he crossed away the fertility defect of the *age-1* mutant, and it still lived long (Johnson *et al.* 1993) [7].

Around the time that Johnson first described *age-1*, I had become very interested in studying ageing. To me, ageing seemed like unexplored territory likely to be full of interesting surprises. I was fascinated by the ‘Hayflick limit’ (Hayflick 1965, 1989) [8,9], which raised the possibility of an intrinsic life timer, and by human progeria diseases (Thomson & Forfar 1950; Brown 1979) [10,11], which suggested that at least some aspects of ageing could be accelerated. Because of my previous scientific experience, I had come to think that there would be universal, evolutionarily conserved regulatory mechanisms for ageing. This had recently been shown to be the case for development, and my laboratory had played a role in this realization, discovering that Hox (Antennapedia-like homeotic) genes patterned the bodies of a much broader spectrum of species than had been anticipated (Costa *et al.* 1988) [12]. In general, this was the time of a great paradigm shift in biology, when we all began to realize that organisms from yeast to humans used highly similar molecular mechanisms, albeit with variation, to carry out the fundamental processes of life. Even if we did not know why we age, ageing is a nearly ubiquitous phenomenon, and something so universal seemed to me likely to be regulated. Furthermore, the remarkable differences in lifespan that one sees between different species could potentially have arisen by changes in regulatory genes. There are long- and short-lived insects, birds and mammals; thus, the rate of ageing appeared to be highly ‘evolvable’. This diversity could arise rapidly if it were driven by changes in regulatory genes, which, like changes in the Hox genes (which, for example, can convert the antennae of flies to legs), could produce large transformations all at once. Eventually I came to hypothesize that there would be some kind of universal mechanism for ageing, controlled by regulatory genes whose activities could be dialled up or down to lengthen or shorten lifespan (Kenyon 1996, 1997) [13,14].

At the time, ageing was generally thought to be a hopelessly intractable, even futile, problem to study. We just wear out; that's it. Fortunately, because of my experience I had come to expect that biological phenomena that seemed to happen haphazardly might well turn out to be controlled by the genes. For example, as a graduate student at MIT, I had worked on a gene that, amazingly enough, was required for UV light to cause mutations (Bagg *et al.* 1981) [15]. Not only was ageing thought to be merely a passive, entropic process, evolutionary biologists had argued forcefully that ageing *could not* be regulated. For example, they felt that mechanisms for regulating ageing would have no way to evolve, as ageing takes place after reproduction. These theories were thought provoking, but to my mind, they had the effect of discouraging searches for regulatory genes. It seemed to me, a molecular geneticist from the outside, that one should keep an open mind and just have a look. So I saw the analysis of ageing as a fantastic opportunity to explore the unknown and perhaps discover something new and important.

I heard Tom Johnson speak at several local genetics and *C. elegans* conferences in the 1980s, as we were both working in California. I told Tom after a meeting at Lake Arrowhead that I thought *age-1* was extremely exciting. I was skeptical of the interpretation that *age-1* mutants lived long only because their reproduction was impaired. The idea that resources saved by not reproducing would automatically be shunted into longevity pathways seemed too simplistic to me. I remember suggesting to Tom that he test the trade-off theory directly, by laser-ablating the reproductive precursor cells of normal worms and asking whether the animals lived longer. Later, I even invited him to our laboratory to use our laser. By this time, I was chomping at the bit to study ageing myself. I decided not to work on *age-1* for two reasons. First, in our friendly *C. elegans* culture, it was not polite to study someone else's gene, and *age-1* belonged to Tom. But mainly, I wanted to carry out my own screen for long-lived mutants, to see what came out.

It had been very easy for me to attract students to my laboratory to study pattern formation, but the situation with ageing was completely different. It took several years to find someone interested in looking for long-lived mutants. At the University of California, San Francisco, CA, where I worked, new graduate students spend a few months ‘rotating’ in each of several laboratories, and I tried to interest these students. However, the ageing field at the time was considered a backwater by many molecular biologists, and the students were not interested, or were even repelled by the idea. Many of my faculty colleagues felt the same way. One told me that I would fall off the edge of the Earth if I studied ageing. However, at last, in the spring quarter of 1992, a wonderful, risk-taking rotation student, Ramon Tabtiang, agreed to come and study ageing.

Ramon had three projects in the laboratory. The first was to do the experiment I described above, to test the reproductive trade-off theory by laser-ablating the cells that give rise to the reproductive system. The second was to screen for long-lived mutants. The third was to ask what effect retinoic acid might have on Hox gene expression in the worm, a project related to my laboratory's general research effort.

The Hox-gene project did not go anywhere, but the two ageing projects went fantastically well. Ramon sat down at our laser-equipped microscope, located newly hatched worms' reproductive precursor cells, and killed them with the laser. The worms grew up and were sterile, but they had a completely normal lifespan. I was delighted. There was no reproductive trade-off. *Caenorhabditis elegans* hermaphrodites contain 959 somatic cells and 2000 germ cells, yet the resources freed up by removing all those germ cells were not redirected to longevity. (This result was consistent with Klass' and Johnson's earlier findings that sterile *fer-15* mutants have a normal lifespan. It was more definitive, however, as *fer-15* mutants, whose sperm are defective, still produce the massive germline and they lay unfertilized oocytes.)

For the mutant screen, we were so lucky that it is still hard to believe. As I mentioned above, to look for long-lived mutants, one needed to control reproduction. To do this, we decided to use a ‘dauer-constitutive’ mutation. Dauer (German for ‘enduring’) is a state of diapause, analogous to a bacterial spore. Dauer formation is essentially a checkpoint that arrests the growth of developing animals at a specific larval stage (an alternative L3 stage) if they encounter low food levels or crowding (figure 1). Dauers are tiny, growth-arrested juveniles that have their own special morphology, do not feed or reproduce, and are quiescent and long-lived. Dauer formation essentially allows the juvenile to outlast harsh environmental conditions before reproducing. When food is restored, dauers resume development and become fertile adults. Only young juveniles can become dauers; once the animals go through puberty and become adults they no longer have this option. Mutations in many genes were known to produce a dauer-constitutive phenotype, in which juveniles enter the dauer state even in the presence of food. (In fact, because dauers are long-lived, Klass had recovered dauer-constitutive mutants in his screen for longevity (Klass 1983) [2].)

**Figure 1.**
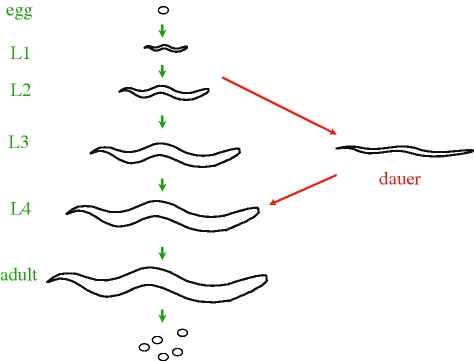
The life cycle of *C. elegans*. Under replete conditions (green arrows), the *C. elegans* hermaphrodite hatches from the egg, passes through four developmental stages (L1–L4), and becomes a fertile adult. Under harsh environmental conditions (red arrows), including low food availability, crowding and elevated temperature, the animals enter the dauer diapause instead of becoming L3 larvae. When environmental conditions improve, the dauers exit from the dauer state to become L4s and then fertile adults.

Our plan was to mutagenize animals that harboured a temperature-sensitive dauer-constitutive mutation. After culturing the animals for several generations at low (non-dauer-inducing) temperature (20°C), so that new mutations could become homozygous via hermaphrodite self-fertilization, we would put individual worms, each a potential long-lived mutant, on separate culture plates and allow each to lay approximately 20 eggs. At this point, we would remove the animal, and allow its progeny to develop past the dauer decision-point. Then we would shift the plates to high temperature (25°C), where, having escaped dauer formation, the worms would continue growth to adulthood. At high temperature, these adults would have progeny, but all the progeny would become dauers, which are small and inconspicuous. Then we would wait until most of the normal adults should have died. If we found plates containing long-lived adults, we could propagate the strain by shifting their dauer progeny back to the low temperature, where they would exit the dauer stage, grow and reproduce.

Many genes affect dauer formation, and, as the entire process is facilitated by high temperature, many dauer-constitutive mutants become dauers at high but not low temperature (Riddle *et al.* 1981; Vowels & Thomas 1992) [16,17]. We chose the mutant *daf-2*(*e1370*) because it was a very ‘tight’ allele: when grown at high temperature all the hatchlings became dauers, and when grown at low temperature, they all behaved like wild type and grew to adulthood. To know when to examine the plates for long-lived mutants, we had to determine when most or all of the control *daf-2*(*e1370*) animals would be dead. Ramon came into my office one day and said: ‘Guess what, they’re not dying'. And we had our first long-lived mutant.

Being a careful scientist, Ramon was not convinced. He suggested that there might be a rogue *age-1* mutation in the background. He finished his rotation and joined another laboratory for his PhD. I went on to measure the lifespans of two other *daf-2* mutants, isolated independently in a different laboratory. They were also long-lived, so the longevity must be caused by *daf-2* mutation. *daf-2* mutants were the most amazing things I had ever seen. They were active and healthy and they lived more than twice as long as normal (Kenyon *et al.* 1993) [18]. It seemed magical but also a little creepy: they should have been dead, but there they were, moving around.

This was a thrilling discovery scientifically, and it was also important from a practical point of view. Now it was easy to attract rotation students to work on ageing. (Several years later, we re-identified *daf-2* in real genetic screens for longevity, first using EMS (Garigan *et al.* 2002) [19] and then RNAi (Hansen *et al.* 2005) [20].)

We characterized the *daf-2* mutants in more detail. For example, we found that they lived long if we cultured them continuously at 20°C, where they grew normally to adulthood. So the animals did not have to grow under dauer-inducing conditions to live long. The simplest interpretation of these results, confirmed later by additional mutant (Larsen *et al.* 1995; Gems *et al.* 1998) [21,22] and RNAi (Dillin *et al.* 2002) [23] analysis, was that severe reductions in *daf-2* activity triggered dauer formation, but milder reductions that permitted growth to adulthood (or severe reductions following the dauer decision point) extended adult lifespan. Later, we showed using RNAi that the wild-type *daf-2* gene acts exclusively during adulthood to affect ageing (Dillin *et al.* 2002) [23], so it acts twice: once to affect dauer formation, which can only take place during development, and again later, to affect ageing.

We also measured the brood size of *daf-2* mutants. We found that at 20°C, the *daf-2*(*e1370*) mutant had 20 per cent fewer progeny than normal. Even though we had shown that loss of the whole reproductive system did not extend lifespan, we wanted to test the significance of this reduced brood size for longevity. A skeptic could argue that *daf-2* mutations caused a *particular type* of change in reproduction that in turn caused longevity. To test this possibility, we killed the four reproductive precursor cells of *daf-2* mutants. The animals still lived long (Kenyon *et al.* 1993) [18]. Thus wild-type animals and *daf-2* mutants had different lifespans in the complete absence of their reproductive systems. Therefore, they had different lifespans for reasons other than differences in fertility. Later, others showed that some *daf-2* mutants had essentially normal reproduction (Larsen *et al.* 1995; Gems *et al.* 1998; Tissenbaum & Ruvkun 1998) [21,22,24], and we were able to uncouple *daf-2's* roles in ageing and reproduction temporally, using RNAi (Dillin *et al.* 2002) [23].

The *daf-2* gene had been known to influence dauer formation since the early 1980s (Riddle *et al.* 1981) [17]. Don Riddle, and also Jim Thomas' laboratory, had shown that the ability of *daf-2* mutants to become dauers required another gene, *daf-16* (Riddle *et al.* 1981; Vowels & Thomas 1992) [16,17]. Of course, I immediately wanted to know whether *daf-16* was also required for the extended lifespans of *daf-2(−)* adults. We still had only rotation students studying ageing. (Other than myself, all of the five authors on our 1993 paper were rotation students, and none joined the laboratory for their PhDs.) I tried to interest someone in testing the role of *daf-16*, but no one agreed, so I did it myself. I found that *daf-16* was completely required for *daf-2* mutants to live long (Kenyon *et al.* 1993) [18]. Thus, these two genes had analogous effects on dauer formation and adult ageing: wild-type *daf-2(+)* prevented wild-type *daf-16(+)* from promoting dauer formation during development and from extending the lifespan of the adult (figure 2). Because it kept animals youthful and extended their lifespans, we nicknamed wild-type *daf-16* ‘Sweet Sixteen’.

**Figure 2.**
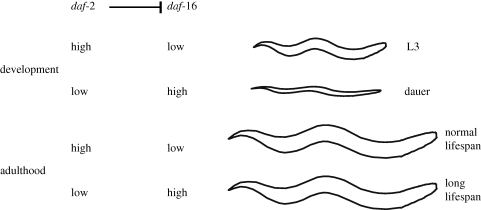
*daf-2* and *daf-16* regulate dauer formation and lifespan. When conditions are favourable during development, wild-type *daf-2* inhibits the activity of *daf-16*, allowing growth to adulthood. Under harsh environmental conditions, *daf-2* activity levels fall, allowing *daf-16* activity to promote dauer formation. During adulthood, reducing *daf-2* activity allows *daf-16* to promote longevity. This genetic pathway was inferred from the mutant phenotypes of *daf-2* and *daf-16*. Long-lived adults carrying relatively weak *daf-2* mutations do not look like dauers, move actively and can be completely fertile. We now know that these genes act exclusively during adulthood to regulate adult lifespan, whereas they act during development to regulate dauer formation. *daf-2* activity in the adult can be influenced by environmental signals, as the pathway can mediate the longevity effects of caloric restriction and it can extend lifespan in response to altered sensory cues. *daf-2* encodes an insulin/IGF-1-receptor that inhibits DAF-16/FOXO transcriptional activity via a conserved protein kinase cascade that acts directly on DAF-16/FOXO. Active DAF-16/FOXO, in turn, influences lifespan by regulating a variety of cell protective and metabolic genes.

In 1992, the dauer pathway was known to comprise three groups of genes (see Riddle & Albert 1997 [25]). One group was later shown to encode a TGF-β signalling pathway. The second group turned out to encode members of a guanylate-cyclase signalling pathway. The third group contained *daf-2* and *daf-16*. We examined the lifespans of animals carrying mutations in genes from these other two groups (Kenyon *et al.* 1993) [18], but none was long lived. Larsen & Riddle also tested the lifespans of a large variety of dauer-constitutive mutants, and, like us, found that *daf-2* mutations increased lifespan, but that dauer-constitutive mutations affecting these other branches did not (Larsen *et al.* 1995) [21].^1^

Our discovery that *daf-2* and *daf-16* affected ageing allowed us to draw some interesting inferences. First, *daf-2* and *daf-16* were known to be regulatory genes, as they regulated dauer formation. Thus, even without knowing the mechanism, the finding that changing known regulatory genes could double lifespan suggested that ageing was subject to regulation. Specifically we said in our paper (about *daf-2* and *daf-16*): ‘Both genes also regulate formation of the dauer larva … Our findings raise the possibility that the longevity of the dauer is not simply a consequence of its arrested growth but instead results from a regulated lifespan extension mechanism that can be uncoupled from other aspects of dauer formation’ (Kenyon *et al.* 1993) [18, p. 461].

Another important aspect of this work is that it was the first clear indication that genes encoding nutrient sensors regulate ageing. The study of dauer formation indicated that, in the presence of food, *daf-2*(*+*) was active and promoted growth to adulthood. Low food was thought to trigger dauer formation by reducing *daf-2* activity. In our paper (Kenyon *et al.* 1993) [18, p. 464], we said: ‘Lifespan in mammals, and to a lesser extent, *C. elegans*, can be increased by food limitation. It is possible that *daf-2* mutations elicit an internal signal also generated by food limitation, which can extend lifespan … It would be interesting to learn whether lifespan extension caused by food limitation requires *daf-16*.’ This does appear to be the case for at least two methods of dietary restriction. Intermittent (every-other-day) feeding extends the lifespan of wild-type animals, but it does not further extend the long lifespan of *daf-2* mutants. In addition, this lifespan extension requires *daf-16* for its full effect (Honjoh *et al.* 2009) [27]. Furthermore, *daf-16* is required for dietary restriction initiated in middle age to extend lifespan (Greer *et al.* 2007) [28]. (Curiously, *daf-16* is not required for lifelong food limitation to extend lifespan (Lakowski & Hekimi 1998) [29] so it is not required for lifespan extension under all conditions of dietary restriction.)

Knowing about *age-1* for so long, I really wanted to learn whether the long lifespan of the *age-1* mutant, like that of the *daf-2* mutant, required *daf-16*. Jenny Dorman, a laboratory technician planning to go to graduate school, addressed this question by building the double *daf-16; age-1* mutant. She (and other laboratories working independently) found that the double mutant was not long lived (Larsen *et al.* 1995; Dorman *et al.* 1995; Murakami & Johnson 1996) [21,30,31]. This was a striking and exciting result. It meant that *age-1* was part of the same pathway as *daf-2* and *daf-16*. All of these genes were likely to be working in a single pathway to influence lifespan. The finding was also crucial from a practical standpoint, as it enabled the subsequent cloning and molecular identification of *age-1*.

The fact that loss of *daf-16*, a gene required for dauer formation, suppressed the long lifespan of the *age-1* mutant, suggested that *age-1*(*hx546*) might be a weak allele of a dauer gene. In fact, a candidate for such a dauer gene existed. This gene, called *daf-23*, behaved like *daf-2* genetically. *daf-23* was being analysed by Gary Ruvkun's laboratory, which had been focusing on the *daf-2/daf-16* branch of the dauer pathway. Gary's laboratory published a beautiful paper about the role of these three genes in dauer formation in 1994 (Gottlieb & Ruvkun 1994) [32]. (Later, they and others showed that *daf-23* mutants were long lived (Larsen *et al.* 1995; Malone *et al.* 1996; Morris *et al.* 1996) [21,33,34].) To me, the exciting thing about *daf-23* was that its map position was close to the map position that Tom Johnson had reported for *age-1* (Johnson *et al.* 1993) [7]. I wanted to test the idea that *age-1* might actually be an allele of *daf-23*, but we still had almost no one working on ageing in the laboratory and therefore could not carry out our own mapping experiments and complementation tests.

The day we learned that the *age-1* mutant's longevity was suppressed by *daf-16* mutations, I called and told my friend Jim Thomas, who studied *daf-16'*s role in dauer formation. I never thought that Jim would start working on *age-1*. However, unbeknownst to me, Jim had found that animals carrying very weak dauer-constitutive mutations could become dauers at 27°C, a very high temperature for worms. Sure enough, *age-1*(*hx546*) mutants became dauers at 27°C. With this obvious phenotype, it was very easy for Jim's group to map *age-1*, by scoring dauer formation rather than lifespan. The position of *age-1* relative to other genes turned out to be slightly different from that reported by Tom (Johnson *et al.* 1993) [7] and exactly the same as that of *daf-23* (Malone *et al.* 1996) [33]. In addition, Jim's laboratory showed that *age-1*(*hx546*) failed to complement *daf-23(−)* mutants. Ergo, they were the same gene. I was disappointed that we were not able to show this ourselves, but at the same time glad that Jim had moved the field forward so quickly. The Thomas laboratory thanked us for ‘communicating unpublished results that motivated us to study the Daf-c phenotype of *age-1’* (Malone *et al.* 1996) [33]. The Ruvkun group also showed that *age-1* and *daf-23* were the same gene (Morris *et al.* 1996) [34].

What kinds of proteins did *daf-2, daf-23/age-1* and *daf-16* encode? During the early 1990s, there was a frenzy of dauer-gene cloning going on in several laboratories (see Riddle & Albert 1997 [25]). In fact, the Ruvkun laboratory was already in the process of cloning *daf-2, daf-23* and *daf-16* when we discovered that *daf-2* and *daf-16* affected ageing. We were of course very interested in learning more about the molecular roles of these genes in ageing. When I first told Gary at a meeting that *daf-2* mutants were long lived, he (like most members of the worm field) did not seem to be interested in ageing. ‘Ageing? You mean, you look at old worms?’ he said. However, being a curious person and fantastic scientist, he soon seemed to have a change of heart. A few weeks later, when we asked him whether he would share sequence information prior to publication so that we could study their molecular roles in ageing, he said no, as he had now become interested in the process. So we needed to clone the genes ourselves. We did not have enough people working on ageing yet to go after both genes, so we chose the more downstream gene, *daf-16*, thinking that it would be closer to the actual mechanism for ageing.

The first gene that Gary and co-workers cloned was *daf-23/age-1*, which turned out to encode a phosphatidyl inositol 3-kinase (PI3-kinase) (Morris *et al.* 1996) [34]. PI3-kinases were known to signal downstream of the insulin and IGF-1 receptor tyrosine kinases, and nowadays, I sometimes hear that the cloning of *daf-23/age-1* was the event that revealed that insulin/IGF-1 signalling affected ageing. However, to my knowledge, no one at the time drew that conclusion. They really could not, as PI3-kinases were part of many different tyrosine kinase and other signalling pathways. (For specific examples, see Auger *et al.* 1989; Varticovski *et al.* 1989; Bjorge *et al.* 1990; Herman & Emr 1990; Sjolander *et al.* 1991; Calabretta & Skorski 1996; Comoglio & Boccaccio 1996; Shimizu & Hunt 1996; Ward 1996; Weiss & Yabes 1996 [35–44].) In his paper, Gary concluded only that *daf-23/age-1* was part of a PI3-kinase pathway (noting presciently that it might involve tyrosine kinase receptors). He did not mention insulin or IGF-1. Likewise, the next year, Tom Johnson wrote ‘*daf-23* has recently been shown to be a PI3-kinase suggesting interesting possibilities in the regulation of the lifespan … ’ (Johnson 1997) [45, p. 18]. Don Riddle, in 1997, wrote: ‘the *daf-2* branch (of the dauer pathway) involves phosphatidylinositol signaling in unknown cells that inhibit dauer formation and limit adult longevity’ (Riddle & Albert 1997) [25, pp. 739–768]. Again, there was no mention of insulin or IGF-1. The nature of this PI3-kinase pathway was revealed later, by the cloning of *daf-2* and *daf-16*.

The DNA sequences of *daf-2* and *daf-16* were determined in 1997. In August, the Ruvkun laboratory reported in *Nature* that *daf-2* encoded the *C. elegans* homologue of the human insulin and IGF-1 receptors (Kimura *et al.* 1997) [46]. This was a stunning finding: hormones, evolutionarily conserved hormones, controlled ageing. The sequence of *daf-2* made sense from the point of view of dauer formation, as IGF-1 and insulin signalling were known to regulate growth and the body's response to nutrients, both of which were integral to the process of dauer formation. However, the idea that inhibiting these essential signalling pathways, which are known for causing diabetes when dysfunctional, could slow ageing and extend lifespan was jarring to some. I remember giving a talk about *daf-2* at a major diabetes meeting, and being told by a rather forceful member of the audience that such mutations would never extend the lifespan of a mammal. They would just cause diabetes. This was Morris White, a world expert in insulin signalling, who later became my friend and went on to report that mouse mutations disrupting the insulin and IGF-1-pathway gene IRS2 extended lifespan (Taguchi *et al.* 2007) [47]. (Interestingly, these mutations also increased blood glucose levels. Reducing insulin/IGF-1 signalling is now thought to extend lifespan, at least in part, by generating a ‘danger signal’ that shifts the animal's physiology towards cell protection and maintenance.)

In 1997, insulin and IGF-1 signalling were known to activate multiple downstream pathways, including PI3-kinase pathways, but the mechanism by which they might affect lifespan was not at all clear. The cloning of *daf-16*, by Gary's and my laboratories, was a major breakthrough. As described above, my laboratory started cloning *daf-16* soon after we found that it affected ageing. The gene was located in a region of the genome that proved difficult to navigate. Both Gary's laboratory and our laboratory initially tried to clone *daf-16* based on its map position. Gary was successful with this approach eventually, while we switched to a different approach, transposon tagging. Gary was the first to sequence the gene, presenting his findings at the *C. elegans* meeting. We gave a poster at the meeting describing our success with transposon tagging, and went on to complete our cloning, independently of Gary's work, soon after. When we were ready to submit our paper, we informed Gary. He quickly wrote his work up, and the two studies were published in December (Lin *et al.* 1997; Ogg *et al.* 1997) [48,49].

The sequence of *daf-16* was incredibly informative. The DAF-16 protein was a forkhead-family (FOXO) transcription factor. This was valuable information for people studying mammalian diabetes and cancer, as it linked a specific transcription factor to mammalian insulin and IGF-1 action for the first time. For ageing, it was monumental. A transcription factor could promote lifespan extension. There was no doubt any more that the ageing process was subject to regulation, and now there was a clear path towards understanding the mechanisms of longevity at the molecular level, at least in part, via the identification and analysis of DAF-16's targets.

Since 1997, the study of insulin/IGF-1 signalling and FOXO (DAF-16) proteins in ageing has exploded. (For additional references, see Kenyon 2005, 2010 [50,51].) Worm lifespan was extended first by six and now by 10-fold, and we now know a lot about the underlying mechanism, which involves DAF-16/FOXO's regulation of different types of cell protective and metabolic genes that appear to act cumulatively to affect lifespan. Many insulin/IGF-1-pathway mutations have been shown to influence lifespan in flies and mice. There are even hints that changes in these regulatory genes can influence lifespan during evolution, an idea that had motivated me to study ageing in the first place: small dogs, which are IGF-1 mutants, live longer than large dogs, and the level of circulating IGF-1 is inversely correlated with lifespan among approximately 30 strains of mice housed at the Jackson laboratories in Maine. At least in worms and flies, FOXO proteins can extend lifespan in response to many inputs (e.g. AMP kinase and Jun kinase activity) not just reduced insulin/IGF-1 signalling. Most exciting are the new links to human longevity. For example, impaired IGF-1 receptor activity has been linked to centenarianism in Ashkenazi Jews, and FOXO DNA variants have been linked to exceptional longevity in Hawaiians of Japanese descent, Californians, New Englanders, Germans, Italians, Ashkenazi Jews and the Chinese. (Though how these FOXO variants affect gene activity, a key question, has not been determined.) Additional transcription factors, such as the heat-shock factor HSF-1, the xenobiotic-response factor SKN-1/NRF and the ER unfolded-protein-response regulator XBP-1 have been shown to contribute to the longevity of *daf-2* mutants. In 1999, the Guarente laboratory discovered that Sir2, later shown to be an NAD-dependent protein deacetylase, can increase lifespan in yeast (Kaeberlein *et al.* 1999) [52], and the roles of sirtuins in ageing have been studied intensively ever since. Additional nutrient, stress and energy sensing pathways, many of which engage in cross-talk with the insulin/IGF-1 pathway, are now known to influence ageing in worms and higher animals, including the TOR pathway, for which life-extending drugs are already available (at least for mice). Perhaps best of all, many long-lived mutants are resistant to age-related diseases, including cancer, heart disease and protein-aggregation disease, suggesting the possibility of forestalling multiple diseases all at once by targeting ageing itself. Along with the many new laboratories that have joined the ageing field, the laboratories of Gary Ruvkun, Tom Johnson, Don Riddle, Pam Larsen and myself have continued to make interesting new contributions. It is all very exciting and wonderful to experience, and of course it is not over yet.

## Endnote

1 Much later, Coleen Murphy's laboratory showed that dauer-constitutive mutations in the TGF-β pathway genes do extend lifespan, but only if the animals are treated with a chemical that prevents the growth of unlaid eggs within the animal (Shaw *et al.* 2007) [26]. As neither Larsen *et al*. [21] nor our laboratory observed a lifespan increase, it must be that animals that would have lived long were killed prematurely by their own progeny.
